# The Effect of Whole-Body Cryotherapy on Exercise Capacity of Healthy Training Men

**DOI:** 10.3390/jcm15156070

**Published:** 2026-08-04

**Authors:** Joanna Beta Łuczak, Sebastian Zduński, Bożena Homola, Monika Kraś, Barbara Myszka, Anna Lubkowska, Waldemar Pluta

**Affiliations:** 1 Rehabilitation Center, Medical Institute of the Ministry of Interior and Administration, Wołoska 137, 02-507 Warsaw, Poland; 2 Centrum Usprawniania Leczniczego, Medical Institute of the Ministry of Interior and Administration, Wołoska 137, 02-507 Warsaw, Poland; 3 Clinical Department of Pulmonology, Internal Medicine, Thoracic Oncology and Transplantology, Medical Institute of the Ministry of Interior and Administration, Wołoska 137, 02-507 Warsaw, Polandmonika.kras@pimmswia.gov.pl (M.K.); barbara.myszka@pimmswia.gov.pl (B.M.); 4 Department of Functional Diagnostics and Physical Medicine, Pomeranian Medical University, 70-111 Szczecin, Poland

**Keywords:** whole-body cryotherapy, exercise tolerance, 6 min walk test, sport

## Abstract

**Objectives**: To analyze the effect of whole-body cryotherapy on exercise tolerance in healthy, training men related to their profession based on the 6 min walk test (6MWT). **Methods**: The study group consisted of 30 individuals aged 24 to 59 years (mean age 39.2; median 38, SD 8.72). The subjects were soldiers from the Special Forces Unit (Military Unit GROM) and officers from the State Protection Service (SOP). All participants underwent a physical fitness assessment using the 6 min walk test: before the study, after two weeks of kinesiotherapy, and after 10 cryotherapy sessions combined with exercise on a stationary bicycle. Kinesiotherapy consisted of 20 min of cycling on a stationary bicycle (cycloergometer) under the supervision of trained personnel (with a load of 50–100 W) from Monday to Friday, from 11 a.m. to 12 noon. Whole-body cryostimulation treatments took place from Monday to Friday from 11 a.m. to 12 noon, entering the cryogenic chamber with a temperature of −130 °C for 2 min, then they went to the gym and rode a stationary bicycle (cycloergometer) for 20 min under the supervision of trained personnel (with a load of 50–100 W). **Results**: Both analyzed factors, i.e., 20 min of stationary cycle ergometer riding and whole-body cryostimulation treatments combined with kinesiotherapy, had a statistically significant effect on improving 6 min walk test results. The obtained results indicate that the impact of the exercise program was slightly greater than the impact of a series of 10 whole-body cryotherapy treatments followed by cycle ergometer riding. **Conclusions**: Both physical activity and cryostimulation had an impact on the physical performance of the subjects, but the obtained results indicate that the impact of exercise alone was slightly greater.

## 1. Introduction

Extensive research exists focusing on non-invasive methods of recovery from exercise-induced muscle damage (EIMD). These include whole-body cryotherapy (WBC), which prevents injuries and counteracts negative inflammatory symptoms during competitive sports [1,2,3,4,5]. Temperatures below −100 °C have a beneficial effect on the bodies of people who exercise regularly.

After a series of 10 whole-body cryostimulation treatments, improvements in motor skills such as balance, flexibility, speed, and dynamic strength of the abdominal muscles were observed. The greatest improvement in equilibrium was noted at a temperature of −160 °C. In turn, the greatest improvement in speed was observed at a temperature of −100 °C [6,7,8]; the number of treatments is important, not the temperature in the chamber proper [9].

In athletes, muscle pain following whole-body cryostimulation is reduced by 80%, along with reduced inflammation and lower levels of muscle cell damage markers [2,3,10]. Research results have shown that a single WBC session has a positive effect on accelerating lactate elimination from the blood after HIT, skin pH, hydration level, and transepidermal water loss (TEWL), but does not significantly alter physiological parameters in young, healthy individuals. Therefore, it is used not only in patients suffering from various diseases but also in healthy individuals as a wellness method [2,11,12,13].

Repeated exposure to WBC has a beneficial effect on mood and anxiety, and improves subjective sleep quality (it extends the duration of slow-wave sleep at night, without affecting other parameters) [14].

## 2. Materials and Methods

Since 2003, the Center for Medical Rehabilitation has been conducting research on the effects of cryostimulation on men working in special military and police units, as well as on SOP officers. The hospital’s Bioethics Committee approved the study (approval number 32/2025) on the effects of whole-body cryotherapy on the physical performance of these individuals. Despite the geopolitical situation and the professional duties performed by the study participants, the project was successfully completed. The framework of the weekly exercise programs, implemented annually by the soldiers and officers, was analyzed by a physiologist and professor at the University of Physical Education in Warsaw. This program, as well as the commandos’ lifestyle, remained unchanged, except that the research elements were incorporated. Unit physicians also conducted periodic check-ups, independent of the tests performed as part of our project. Participation in the study was completely voluntary; each participant signed an informed consent form and was assessed by a physician. The group initially consisted of 35 trained men, 30 of whom completed the study. They ranged in age from 24 to 59 years (mean 39.2 years, median 38 years) and participated in competitive sports as part of their profession. The body weight of the commandos ranged from 71 to 117 kg (mean 88.4 kg, median 86 kg; SD 10.39 kg), and their height ranged from 168 to 193 cm (mean 181.4 cm, median 182 cm; SD 5.63 cm). The study was conducted at the Ministry of Interior and Administration’s Medical Rehabilitation Center from Monday to Friday (excluding Saturdays and Sundays) for two weeks. The men rode a stationary cycle ergometer for 20 min under the supervision of trained personnel (with a load of 50–100 W), which served as the control group in the project. Then, for the next two weeks, the same group underwent 2 min of whole-body cryostimulation in a chamber at −130 °C, and then rode a stationary cycle ergometer for 20 min, similar to the previous two weeks. Whole-body cryotherapy involves entering a room with a temperature below −100 °C and then undergoing kinesiotherapy. Hence, the idea of incorporating a stationary cycloergometer was born to maintain the methodology of the treatment and ensure a clear cryogenic result. The study group had their measurements taken on Saturdays. Before, during (after two weeks), and after the project, exercise capacity was assessed using a 6 min walk test. The test was conducted on a flat, straight corridor in the Hospital’s Department of Internal Medicine, Pulmonology, Oncology, and Transplantology, where a 30 m distance was measured with a laser. It appears that this is why the distance was precisely calculated. For each man, the predicted value and the lower limit of normal were calculated individually according to Enright:

distance (predicted value) = (7.57 × height [cm]) − (5.02 × age) − (1.76 × body weight [kg]) − 309 m

lower limit of normal = predicted value − 153 m.

In the first stage of the analysis, statistical indices (mean, median, maximum, minimum, mode, variance, and standard deviation) were calculated for each of the three series of 6 min test results conducted during the study. Participants’ results were compared with their individually determined predicted values. In the second stage of the analysis, the distribution of 6 min test results was assessed. The normality of the distributions was verified using the Shapiro–Wilk test, recommended for small sample sizes (*n* < 100). Microsoft Excel and tables of coefficients were used for calculations. For the assumed significance level, the critical value of the test was 0.927. In all cases analyzed, there was no reason to reject the null hypothesis, justifying the use of parametric methods in further analysis. Because each participant was tested three times and subsequent measurements were from the same individuals, a one-way repeated-measures analysis of variance (ANOVA) was used. This method allows for the assessment of the effect of time and subsequent intervention stages on changes in the mean values of the analyzed parameter, taking into account the relationship between subsequent measurements performed in the same participants. In addition to assessing statistical significance, effect sizes were also calculated. For the analysis of variance, the partial eta squared coefficient (partial η^2^) was determined, which describes the contribution of the analyzed factor to the total variability of the results. For pairwise comparisons, the Cohen’s d coefficient for dependent samples was calculated, allowing for the assessment of the practical significance of the observed changes regardless of the sample size. Statistical analysis of the obtained results was performed using Microsoft Excel. A statistical significance level of α = 0.05 was adopted for all tests. 

## 3. Results

**Figure 1:**
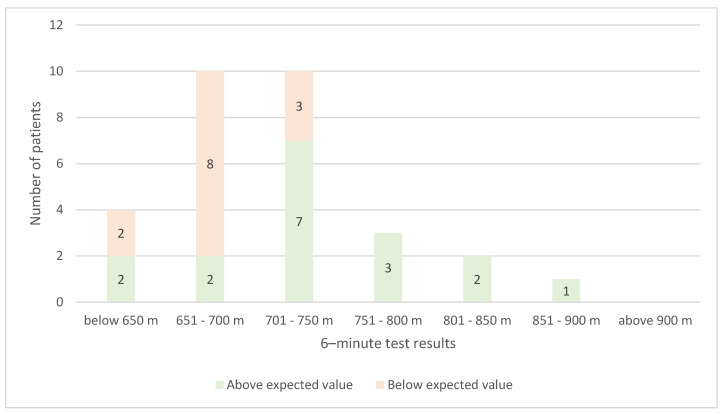
Comparison of the 6 min walking test results at the beginning of the study, taking into account the predicted values.

At the beginning of the study, 17 subjects achieved test results 6 min above their individual predicted values, while 9 subjects achieved results below their individual predicted values (Table 1, Figure 1). The mean result was 717.67 m, with a median of 715.00 m and a standard deviation of 63.65 m.

**Figure 2:**
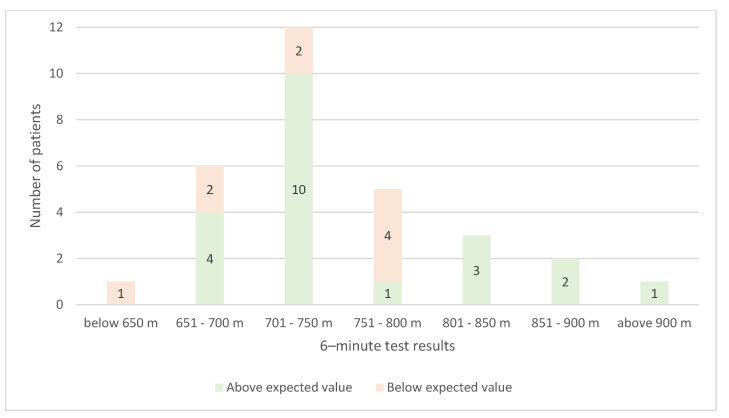
Comparison of the 6 min walking test results after the first stage of the study, taking into account the predicted values.

After the exercises (stage I of the study), the number of subjects achieving at least the predicted value rose to 21, while 9 subjects achieved results 6 min below (Table 1, Figure 2). The average result was 754.17 m, with a median of 750.00 m and an SD of 67.26 m.

After 10 whole-body cryostimulation treatments (stage II of the study), more subjects achieved at least the predicted value (29 subjects), while the number of subjects who did not achieve the predicted value decreased (1 subject). The average result was 793.67 m, with a median of 780.00 m and an SD of 74.67 m.

**Figure 3:**
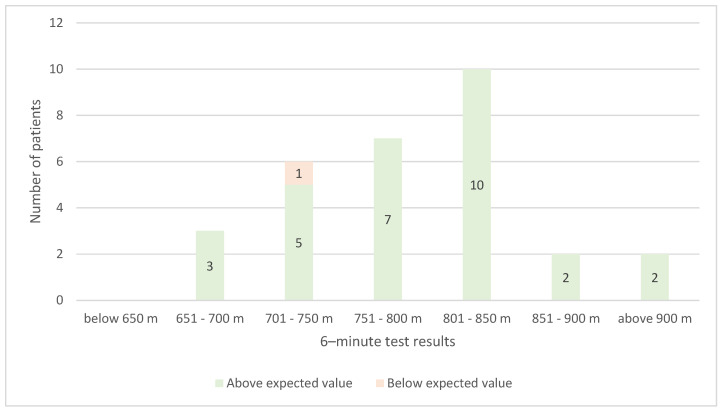
Comparison of 6–Minute Walking Test results after Stage II of the study, taking into account predicted values.

**Figure 4:**
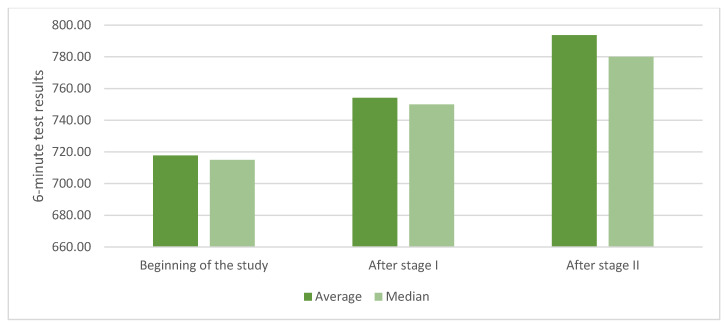
Comparison of the mean and median 6-minute walking test results during the study.

Throughout the study, systematic improvements in 6 min test results were observed, both after implementing the exercise program and after using the cryogenic chamber combined with cycling. Following the exercise program, all key statistical indicators increased: the arithmetic mean (by approximately 5.1%), the median (by approximately 4.9%), the minimum (by approximately 2.4%), the maximum (by approximately 3.0%), the mode (by approximately 3.3%), and the standard deviation (by approximately 5.7%). Similarly, the values of key statistical indicators also increased after the use of the cryogenic chamber combined with kinesiotherapy: the arithmetic mean (by approximately 5.2% compared to the post-exercise measurement and by approximately 10.6% compared to the initial measurement), the median (by approximately 4.0% and 9.0%, respectively), the minimum value (by approximately 6.3% and 9.0%), the maximum value (by approximately 6.5% and 10.0%), the mode (by approximately 8.0% and 17.4%), and the standard deviation (by approximately 11.0% and 17.3%) (Figure 3 and Figure 4, Table 2).

Overall, a progressive improvement in the 6 min walk test performance was observed throughout the whole study. The proportion of participants achieving results at or above their expected values increased from 56.7% at the beginning of the study, to 70.0% after the first stage of the study, ending at 96.7% after completion of the second stage of the study (Table 1). A steady increase in statistical indicators like the mean, median, minimum, maximum, and modal values of the walking distance was observed with each measurement (Table 2).

The Shapiro–Wilk test indicated no evidence against the assumption of normality for any of the three measurement time points (significance level α = 0.05), supporting the use of parametric methods. Repeated-measures ANOVA demonstrated a statistically significant effect of time on the 6 min walk test results (*p* < 0.001), with a very large effect size (partial η^2^ = 0.583). Post hoc comparisons with Bonferroni correction showed statistically significant differences between all consecutive measurements (*p* < 0.001) (Figure 4). Large effect sizes were observed for the exercise program (Cohen’s d = 1.13), the cryostimulation stage combined with cycling (d = 0.93), and the overall intervention (d = 1.27).

These findings indicate that participants achieved progressively better results over the course of the rehabilitation program. However, because all participants underwent the same sequence of interventions and no control group was included, the present analysis does not allow the independent effects of exercise and whole-body cryostimulation to be separated or causal conclusions regarding the additional benefit of cryostimulation to be drawn. The observed improvements should therefore be interpreted as changes associated with the combined rehabilitation protocol applied in this study.

## 4. Discussion

The 6 min walk test (6MWD) is often used to objectively assess exercise capacity in both healthy and sick individuals, including children, adolescents, adults, the elderly, and exceptionally athletic individuals [15,16,17,18,19,20,21,22,23,24,25]. The effect of supplementation with fruit juices rich in polyphenols (PP) on the results of this test has even been studied, as has the effect of rapid ascent to very high altitudes of 2900 m and 5000 m [25,26]. Such a wide application requires reliable performance, but does not require complicated equipment or specialized technical knowledge. It involves walking as far as possible along a 30 m corridor with minimal traffic for 6 min. The main assessment parameter is the 6 min walk distance (6MWD), measured in meters. Factors influencing the result include the track layout (continuous or straight), track length, oxygen saturation and ease of movement, learning effect, and verbal encouragement. Additionally, derived indicators such as the distance-desaturation product (the product of the oxygen saturation nadir and the walking distance) can be analyzed [15].

The gait itself can also be analyzed during the test: step length, walking speed, double support, step duration, stance phase, step duration asymmetry, step duration variability, foot strike, and toe-off [16]. Furthermore, step count can be predicted with moderate predictive power from demographic and anthropometric variables such as height, weight, ethnicity, age, and developmental stage [27]. This test can detect comorbid chronic respiratory diseases, including cardiovascular disease, frailty, sarcopenia, and cancer [15]. To obtain reliable and reproducible results, careful attention must be paid to the methodology. Although guidelines for performing the 6MWT have been established, it is not always possible to perform this test in every location due to physical and spatial constraints.

Evidence suggests that the conditions during the 6MWT significantly impact the results and, therefore, their reliability. Using a ground-based path covering the longest possible linear distance appears crucial to ensuring high reliability [28]. A short out-and-back path leads to lower results than longer distances. The walking distance covered on a rectangular path or a 10 m figure-eight path is greater than that achieved by walking back and forth on a five- to ten-meter path. The efficiency achieved on a treadmill is lower than that achieved by walking on the ground [29].

Simplified field tests are gaining popularity in assessing functional capacity, but direct comparisons of functional performance and physiological responses between the 1 min sit-to-stand test (1MSTST) and the 6 min walk test (6MWT) remain limited [30,31]. Maximum walking distances have been compared across different walking distances: 30 m and at intervals below, specifically, 10, 15, 20, and 25 m [32]. The 6 min stride test is a reliable and valid test. In the National Emphysema Treatment Trial (NETT), the difference between walking on a straight path with turns and walking on a rectangle was 33.5 m, or 10% in favor of the latter. Research was conducted to establish a reference equation for predicting total six-minute walking distance (6MWD) for healthy adults aged 40 to 80 years. Oxygen saturation (SaO22), pulse rate, and degree of dyspnea (Borg scale) were determined before and at the end of the walk. The median distance was 576 m for men and 494 m for women [22]. The resulting sex-specific regression equations explained approximately 40% of the variance in distance traveled by healthy adults: for men 6MWD = (7.57 × height cm) − (5.02 × age) − (1.76 × body weight kg) − 309 m, and for women 6MWD = (2.11 × height cm) − (2.29 × body weight kg) − (5.78 × age) + 667 m. These reference equations can be used to calculate the percent predicted 6MWD for individual adult patients performing the test for the first time, when using a standardized protocol. The American Journal of Respiratory and Critical Care Medicine issued a reference for the six-minute test in healthy adults; subsequently, errata and corrections were introduced [22].

Considering adults, there are many formulas for calculating the predicted value. One suggestion for people aged 20–50, where the tests were performed in a 30 m corridor, is the Chett formula for women and men: Women 6MWD (m) = 518.53 + (1.25 × height) − (2.816 × age) − 39.07. Whereas Gibbons gives: Women 6MWD (m) = 868.8 − (age × 2.99); Men 6MWD (m) = 868.8 − (age × 2.99) − 7.47 [29].

Normal saturation values are 95–98% (SpO_2_ ≥ 93% is the so-called broad norm). A saturation below 90% indicates respiratory failure. Despite the publication of several formulas for predicted values, no clear interpretation scheme for 6MWD expressed as a percentage of the predicted value has been developed. A significant reduction in walking distance should be considered a reduction in 6MWD < 82% of the predicted value or below the lower limit of normal when using a formula enabling calculation of this variable. Clinically significant desaturation should be considered a value ≥ 4% (a reduction in SpO_2_ compared to the resting value) or a reduction in SpO_2_ to below 88% or 90% [33].

## 5. Conclusions

Both analyzed factors, i.e., 20 min of stationary cycle ergometer riding under the supervision of trained personnel (with a load of 50–100 W) and whole-body cryostimulation treatments combined with kinesiotherapy, had a statistically significant effect on improving 6 min walk test results. The obtained results indicate that the impact of the exercise program was slightly greater than the impact of a series of 10 whole-body cryotherapy treatments followed by cycle ergometer riding, as confirmed by the Cohen’s d effect size coefficients.

However, it should be emphasized that both effects were significant, demonstrating the significant importance of both physical activity and cryostimulation in improving the subjects’ performance. I did not find any studies in the literature on the effect of cryotherapy on the physical performance of healthy men training for their professions; hence, the idea to conduct such studies.

## Figures and Tables

**Table 1:** Comparison of 6-minute walking test results with predicted values.

Result	Beginning of the Study		After Stage I of the Study		After Stage II of the Study	
	n	%	n	%	n	%
Result Above Predicted Value	17	56.7%	21	70.0%	29	96.7%
Result Below Predicted Value	13	43.4%	9	30.0%	1	3.3%

**Table 2:** Comparison of basic statistical indicators of the 6–minute walking test results.

Parameter	Study Start	After Stage I of the Study	After Stage II of the Study
Arithmetic Mean $\bar{x}$	717.67	754.17	793.67
Median me	715.00	750.00	780.00
Minimum Value MIN	615.00	630.00	670.00
Maximum Value MAX	900.00	930.00	990.00
Range [m]	615.00–900.00	630.00–930.00	670.00–990.00
Modal value D	690.00	750.00	810.00
Variance $s^{2}$ [m^2^]	4051.26	4524.28	5575.75
Standard Deviation s [m]	63.65	67.26	74.67

## Data Availability

Medical Institute of the Ministry of Interior and Administration.
